# TECHNOLOGY TO SUPPORT AGING ADULTS: FUTURE APPLICATIONS

**DOI:** 10.1093/geroni/igac059.862

**Published:** 2022-12-20

**Authors:** Sara Czaja, Walter Boot, Neil Charness, Wendy Rogers, Joseph Sharit

**Affiliations:** Weill Cornell Medicine, New York City, New York, United States; Florida State University, Tallahassee, Florida, United States; Florida State University, Tallahassee, Florida, United States; University of Illinois Urbana Champaign, Champaign, Illinois, United States; University of Miami, Davie, Florida, United States

## Abstract

The aging of the population is projected to continue for the upcoming decades. Thus, innovative strategies are needed to support current and future cohorts of older adults. In this regard, there are exciting developments in technology that have the potential to meet the needs of population aging. This summary presentation will discuss emerging technology applications such as developments in artificial intelligence, sensing technologies, and robotics that can be used to foster everyday activities, cognitive, physical, and emotional health. A framework will be provided to characterize current and emerging technology applications for older adults within life domains. Current research examining the efficacy, feasibility, and acceptability of these technologies with older adult populations will be also summarized. Recommendations for needed future research in the aging and technology domain will also be provided to ensure that the emerging technology applications are designed to meet the needs, abilities, and preferences of aging adults.

